# CSF from Parkinson disease Patients Differentially Affects Cultured Microglia and Astrocytes

**DOI:** 10.1186/1471-2202-11-151

**Published:** 2010-11-29

**Authors:** Mya C Schiess, Jennifer L Barnes, Timothy M Ellmore, Brian J Poindexter, Kha Dinh, Roger J Bick

**Affiliations:** 1 Department of Neurology, University of Texas Medical School at Houston, Houston, Texas 77030, USA; 2 Department of Neurosurgery, University of Texas Medical School at Houston, Houston, Texas 77030, USA; 3 Department of Pathology and Laboratory Medicine, University of Texas Medical School at Houston, Houston, Texas 77030, USA

## Abstract

**Background:**

Excessive and abnormal accumulation of alpha-synuclein (α-synuclein) is a factor contributing to pathogenic cell death in Parkinson's disease. The purpose of this study, based on earlier observations of Parkinson's disease cerebrospinal fluid (PD-CSF) initiated cell death, was to determine the effects of CSF from PD patients on the functionally different microglia and astrocyte glial cell lines. Microglia cells from human glioblastoma and astrocytes from fetal brain tissue were cultured, grown to confluence, treated with fixed concentrations of PD-CSF, non-PD disease control CSF, or control no-CSF medium, then photographed and fluorescently probed for α-synuclein content by deconvolution fluorescence microscopy. Outcome measures included manually counted cell growth patterns from day 1-8; α-synuclein density and distribution by antibody tagged 3D model stacked deconvoluted fluorescent imaging.

**Results:**

After PD-CSF treatment, microglia growth was reduced extensively, and a non-confluent pattern with morphological changes developed, that was not evident in disease control CSF and no-CSF treated cultures. Astrocyte growth rates were similarly reduced by exposure to PD-CSF, but morphological changes were not consistently noted. PD-CSF treated microglia showed a significant increase in α-synuclein content by day 4 compared to other treatments (p ≤ 0.02). In microglia only, α-synuclein aggregated and redistributed to peri-nuclear locations.

**Conclusions:**

Cultured microglia and astrocytes are differentially affected by PD-CSF exposure compared to non-PD-CSF controls. PD-CSF dramatically impacts microglia cell growth, morphology, and α-synuclein deposition compared to astrocytes, supporting the hypothesis of cell specific susceptibility to PD-CSF toxicity.

## Background

Evidence of increased levels of specific cytokines and growth factors within nigrostriatal dopamine regions of the brain in Parkinson's disease (PD) patients, has led to the belief that PD is the result of immunological responses that promote an increased synthesis and release of proinflammatory cytokines [1-3]. These cytokines have been shown to affect the quantity and distribution of intracellular proteins such as α-synuclein in cultured microglia [4]. The exact function of α-synuclein is unknown. However, there is evidence supporting a vesicular, pre-synaptic role for α-synuclein in the dopamine transporter system [5-7]. This normally soluble protein is recognized to be a large component of the Lewy body, the pathologic hallmark of the disease. What promotes the formation of Lewy body inclusions is poorly understood, but it has been proposed that this is a protective pathway in response to failed mechanisms, such as aggresome degradation of dysfunctional protein [8]. The Lewy body, and its precursor the Lewy neurite, have been reproducibly traced through the CNS, resulting in a progressive and predictable pattern of involvement leading to the staging of sporadic Parkinson's disease and the clinical correlation of symptoms with neuroanatomical localization [9].

The majority of investigations regarding the pathogenic mechanisms underlying cell death in PD have emphasized post-mortem studies, genetically altered animals and neuronal cultures, but a role for non-neuronal cells in the pathoetiology of PD by investigations into glial cell line responses, may lead to a better understanding of the role of α-synuclein in cell-cell communication and the neuron-glia relationship in PD. Our previous work with cultured microglia cells showed that after exposure to specific cytokines, α-synuclein was both redistributed and increased in content, with the cells becoming prone to enter cell death pathways [4]. We therefore treated cultured human microglia cells with PD-CSF to see if we could produce similar results. The cells responded dramatically, exhibiting reduced growth, a loss of adhesion, and eventual necrotic death. Given these findings with cultured microglia, we postulated that effects in other glial cell lines such as astrocytes might be similar to those in microglia. Astrocytes have not been investigated to as great a degree as other cell types, despite having critical signaling and support roles in the CNS. Astrocytes are known to be involved in protection against neurodegeneration, and display α-synuclein immunoreactivity in cases of sporadic PD [10-13]. Therefore, the purpose of this study was to continue a line of research exploring the effects of PD-CSF and disease control CSF on cultured glial cells, while comparing outcomes with microglia and astrocytes in terms of resiliency and protein aggregations and distributions. These two cell types differ in their functions in the CNS and in their origins, microglia being derived from bone marrow and functioning primarily as phagocytic neuroprotective first responders, whereas astrocytes are multi-task cells derived from neurectoderm, with functions ranging from metabolic buffers and detoxifiers, to providers of endothelial support and scar formation. We hypothesized that these two functionally diverse cell lines would both succumb to PD-CSF treatments, but different responses would occur in protein losses/changes and ability to overcome these challenges. Using not only PD-CSF, but also non-PD disease control CSF and no-CSF treatments, expands our limited knowledge of cellular responses in PD. Previous studies have shown the 'toxicity' of PD-CSF on adrenal medulla tumor cells and retardation of cell growth [14]. Since 1995, it has been known that PD-CSF contains factors which result in dopaminergic neuron distress and growth inhibition [15,16]. However, the role of non-neuronal cells in disease progression has been studied to a lesser degree, even though their roles in signaling and neuron support are well established.

## Results and Discussion

### Cultured microglia cells growth patterns

**Figure 1:**
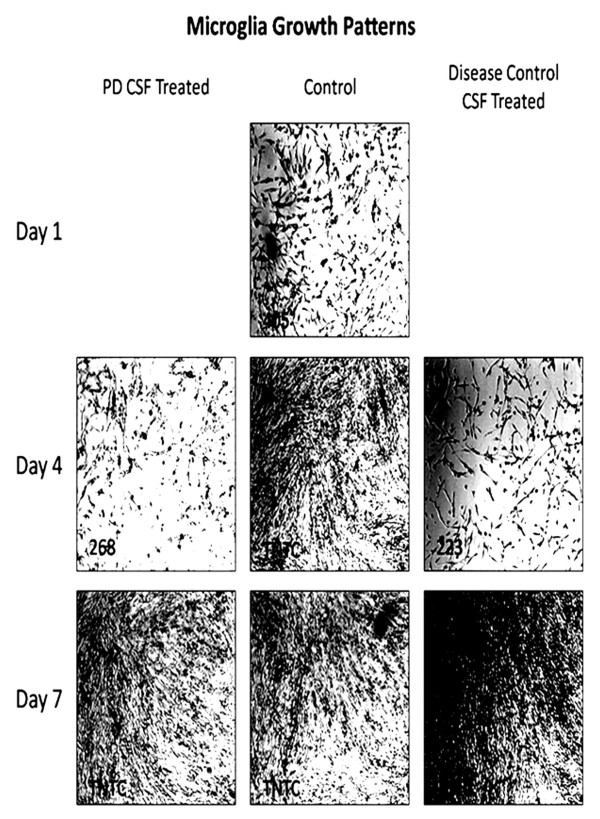
**Photographs of cell growth of cultured microglia showing that by day 4, controls had grown to confluence, whereas PD-CSF treated cells had not**. Disease (neurological) control CSF treated cells had a reduced growth rate. After addition of fresh growth medium (Day 7) all cell groups showed substantial growth. Cell count per frame is in lower left corner; TNTC = too numerous to count. Magnification ×100.

Microglia cells showed a reduced rate of growth following PD-CSF treatment, as well as diverse morphological changes that included cellular blebbing, but these observations were not apparent in disease control CSF treated or control (no-CSF) microglia cultures. Figure 1 demonstrates that by day four control cells had grown to confluence, whereas PD-CSF treated microglia were sparse and non-confluent. Disease control CSF treated cells had a reduced growth rate, but overt changes in cell morphology were not apparent. By day seven, after the addition of fresh culture medium, all cell groups showed substantial renewed growth and viability (Magnification ×400).

### Cultured astrocytes cell growth patterns

**Figure 2:**
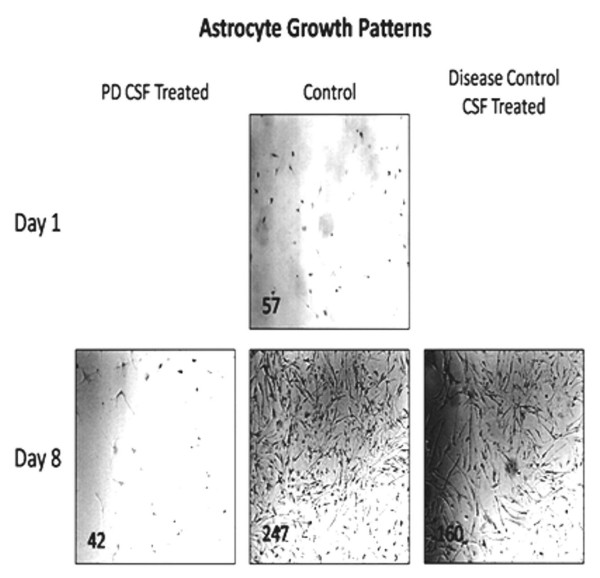
**Photographs of astrocyte cultures in which diverse morphology changes were not evident between the PD-CSF, control and diseases control CSF groups**. With the addition of disease (neurological) control CSF, a higher growth rate was seen than that in PD-CSF treated cells, but a lower growth rate than in the control (no-CSF) treated cultures. Cell count per frame is in lower left corner; TNTC = too numerous to count. Magnification ×100.

Figure 2 illustrates variable and minimal changes in morphology of the astrocyte cultures. PD-CSF treated astrocytes displayed a slower growth rate than the microglia. However, when disease control CSF was added to astrocyte cultures, the growth rate was much higher than that in the PD-CSF treated cells, but slower than the no-CSF treated cultures. Cell growth for cultured astrocytes continued at a slower rate following exposure to PD-CSF even after addition of fresh medium, which did support some increased growth (Magnification ×400).

### α-synuclein distribution in microglia

**Figure 3:**
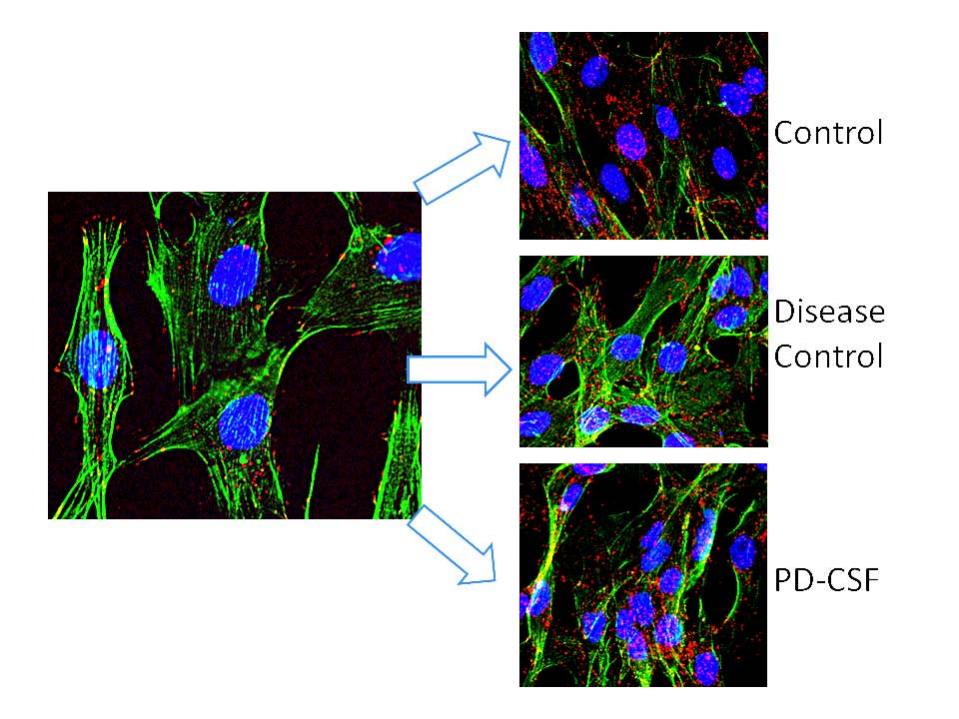
**Fluorescence deconvolution microscopy images showing an increased density of α-synuclein in microglia following PD-CSF treatment, but not in neurological disease control CSF and no-CSF controls**. Red = α-synuclein; blue = DAPI (nuclei); green = filamentous actin. Magnification ×600.

As seen in Figure 3 the intracellular content of α-synuclein following PD-CSF treatment, compared to disease control CSF treated and no-CSF controls, was significantly increased (p < 0.05 for all comparisons; 1575 ± 1225 v 513 ± 235 v 618 ± 197, PD v control disease v control; pixel densities, n = 8 experiments). However, what is of note is that α-synuclein protein increases were in a somewhat random, intracellular locale, not along the periphery of the cell and clustered to the nucleus (Magnification ×600).

### α-synuclein density in astrocytes

**Figure 4:**
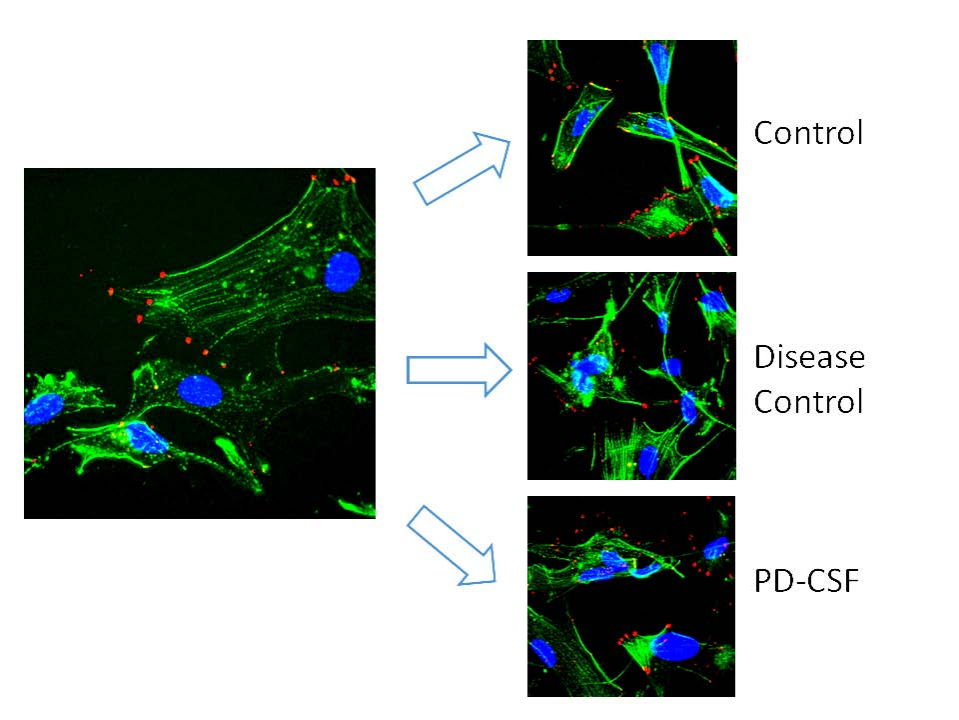
**α-Synuclein density in astrocytes, α-Synuclein distribution patterns were not different between treatment groups**. Red = α-synuclein; blue = DAPI (nuclei); green = filamentous actin. Magnification ×600.

In contrast to microglia cultures, Figure 4 shows that the α-synuclein density in cultured astrocytes was variably affected, with no discernable increases in protein content following various CSF treatments (Control 333 ± 264; disease-control 480 ± 324; PD-CSF treated 511 ± 261; pixel densities, n = 8 experiments). Unlike the microglia cells, the α-synuclein seen at the cell extensions, was minimally disrupted (Magnification ×600).

### α-synuclein content in treated microglia cells

**Figure 5:**
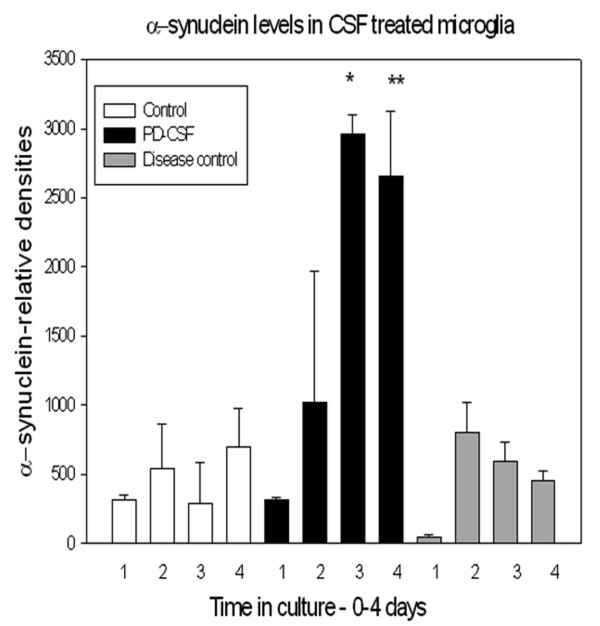
**Histogram to show changes in α-synuclein content of cultured microglial cells for all treatment groups over consecutive days 1-4**. Treatment with PD-CSF resulted in significant increases of α-synuclein content Day 3 *, Day 4 **; (p < 0.02) when compared to control and neurologic disease control CSF treated cells (n = 8).

Relative densities of α-synuclein content in cultured microglia over time (consecutive days 1-4), between the three treatment groups are shown in Figure 5. PD-CSF treatment resulted in a rapid, significant increase in α-synuclein content, compared to both the no-CSF control and disease control CSF treated cells by days 3 and 4 (p ≤ 0.02).

### α-synuclein content in treated astrocytes

**Figure 6:**
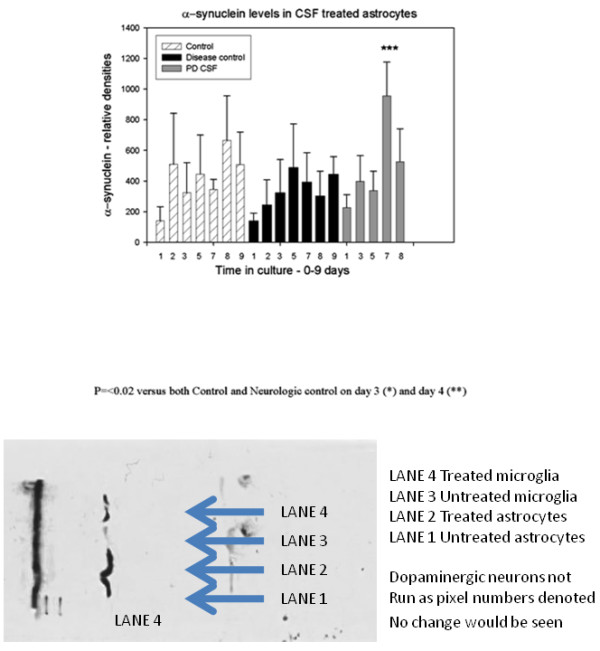
**Histogram demonstrating changes in α-synuclein content of cultured astrocytes of all treatment groups over the course of the experiment**. On day 7 there was a significant (p < 0.05) increase in α-synuclein content in the PD-CSF treated astrocytes, compared to the neurological disease control CSF treated cells, but all cells showed a trend to a return to baseline of α-synuclein content (n = 8). Western blot of protein levels is included for comparison.

In contrast to microglia, Figure 6 illustrates that treated astrocytes showed modest increases in α-synuclein content, with a tendency to return to pre-treatment levels without further additions of CSF. Only the PD-CSF treated cells revealed significant increases in α-synuclein, and this was much later, on day 7, than in microglia (p ≤ 0.05). These later increases were not sustained and disappeared over the next 2-3 days. These changes are also shown in the Western blot.

### α-synuclein distribution in modeled microglia and astrocyte cells

**Figure 7:**
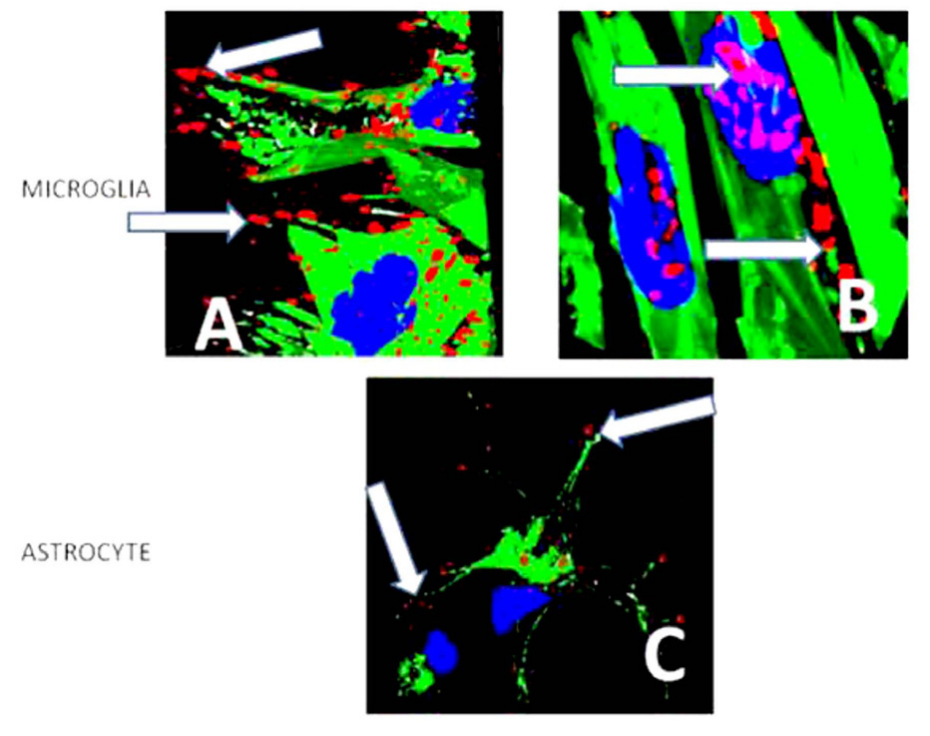
**Microglia models showing α-synuclein locations**. 3D models of stacked deconvoluted acquisitions to show the initial peripheral localization of α-synuclein in microglia (Image A), which becomes aggregated and peri-nuclear or juxta-nuclear following PD-CSF treatment (Image B). Astrocytes (Image C), still demonstrate peripheral α-synuclein and minimal aggregation, even after seven days of treatment (Magnification × 900).

Figure 7 shows three models of cultured cells. Panels A and B are images of microglia before and after treatment with PD-CSF to definitively reveal the changes in both content and distribution of the α-synuclein. Note that treatment with PD-CSF causes the normal peripherally located protein (red; white arrows, Image A) to aggregate and become peri- or juxta-nuclear in location corresponding to the increased cellular content shown in Figure 6 (Image B). However, the astrocyte shown (Image C) still retains its component α-synuclein at a constant level and predominantly in the normal, peripheral and cytoplasm distribution patterns, even 7 days after PD-CSF treatment (blue = nucleus, DAPI probe; Green = Phallicidin-f actin; red=α-synuclein; model constructed from >30 sections: Magnification ×900).

## Discussion

Based on our previously reported results with cultured microglia and cytokines, we postulated that exposure of these cultured cells to PD-CSF, which has been reported to contain cytokines, would give similar results [1]. As a comparison to microglia, which play a critical role in neurodegenerative disease and may well be an integral part of the immunologic cascade that occurs in PD and possibly Alzheimer's disease, we chose to culture astrocytes since they have been shown to play a protective, anti-inflammatory and signaling role in the brain [17,18,10].

We hypothesized that neuro-toxic effects from PD-CSF exposure would be specific to the individual cell types given that they have different roles in the CNS [4,19].

Interest in comparing these two essential CNS cell types was piqued by reports of microglia-astrocyte interactions in neurodegeneration and the lack of understanding of the role of astrocytes in Parkinson's disease [20,21]. We therefore undertook a localization-quantification direction in our investigations to carry out cell comparison studies, and found that there were indeed different outcomes in our two cell lines with regard to protein content and cell growth responses following treatments with PD-CSF, a fluid known to be toxic in cell cultures [14,16].

Our previous research reported the effects of cytokines on both protein aggregations in cultured cells and resulting cell viability. Results revealed that glial cells were not only deleteriously affected by sustained and high-dose cytokine treatments, but that these changes were continuous by exposure to high doses, in regard to both protein localizations and protein content [4,19]. We therefore wanted to determine the effects of PD-CSF treatments on cultured glial cells, while comparing outcomes to changes seen in cultured astrocytes.

While both cell types showed somewhat similar alterations in growth patterns, with astrocytes growing at a slower rate, dramatic changes were seen in glial cell morphology, with evidence of cellular blebbing and a much decreased growth rate, suggesting their greater susceptibility to agents such as CD14 or secreted alpha-synuclein [18,19]. Furthermore, the two cell types revealed contrasting outcomes when α-synuclein distributions were compared following exposure to PD-CSF. Astrocytes revealed minimal changes in distributions of α-synuclein, while microglial cells exhibited a marked change from a diffuse distribution pattern throughout the cytoplasm and along the plasma membrane, to a dense, peri-nuclear aggregation. Whereas astrocytes did not reveal defined changes in α-synuclein protein densities, glial cells did demonstrate increasing amounts of the protein after PD-CSF treatment, amounts that were significantly higher than those found in both control (media only) and disease control CSF treated cells on day 4 post-treatment. Additionally, these increases in α-synuclein content were concurrent with reduced glial cell growth, suggesting that the different outcomes might reflect diverse roles for α-synuclein in the two cells types and lending support to reports of over expression of α-synuclein by genomic multiplications leading to Parkinsonism [21-25]. Excess α-synuclein causes deleterious effects in cultured glia, and infers disruptions in cellular machinery involved in membrane trafficking, chaperon-mediated autophagy, or via the production of excessive amounts of reactive oxygen species leading to oxidative damage [26,27]. In contrast, astrocytes appear to be more resilient to PD-CSF assault and somewhat immune to protein aggregations, thereby suggesting that astrocyte protection of neurons might be sustained under attacks from such factors as soluble α-synuclein, astrocyte originating prostaglandins and other noxious compounds [28-30]. These findings also give support to the possibility that astrocytes, in which α-synuclein plays a role in fatty acid uptake and trafficking, suffer dire consequences to their membrane functionality, rather than in their signaling role [23].

Due to the results of this study revealing an apparent resiliency of astrocytes, our future research will include a focus on changes in cell adhesion properties as a cause of neurodegeneration, the role of glial derived neurotrophic factor (GDNF) in PD and cellular protection, and the co-culturing of CNS cells to display neuroprotective effects of GDNF and the proactive role of astrocytes in neurodegeneration [31,32]. Isolation and identification of circulating factors involved in chronic neurological diseases is an ongoing task, but the roles of specific cells and their diverse reactions to PD-CSF treatments revealed by these comparative studies are important in not only understanding the interactions between cells in neurodegeneration, but possibly by refocusing research to pathways and mechanisms that can halt the loss of dopaminergic neurons by looking at other cell types. Sporadic PD has a variable phenotypic expression and rate of progression, no biological marker exists and as such the diagnosis is made clinically, being solely dependent on symptoms and signs of motor dysfunction from striato-nigral failure [33]. Nonetheless, our PD patient population in this study was at least seven years post their PD diagnosis and in the off medicine state when CSF was obtained, placing them uniformly in a moderately severe stage of the disease. In order to exclude non-specific effects, we used neurological disease controls or non-PD CSF for comparison, samples that were harvested and stored similarly.

## Conclusion

To summarize, these findings show that not all cell types in the CNS react in a similar manner to exposure to CSF from PD patients. The data suggest that a constituent, or constituents, specific to PD-CSF, leads to cell growth retardation and exerts specific and possibly unique effects on α-synuclein distribution and densities in multiple cell types, thus disrupting multiple pathways.

Previous results combined with those presented here, illustrate the important role α-synuclein plays in the pathology of PD and the profound changes that occur due to exposure to PD-CSF and/or cytokines [4,20]. Apparent recovery of the microglia over time suggests that the cells appear to flirt with a death pathway, but are able to overcome the low-dose/initial insult of PD-CSF and cytokine treatments, and return to a robust growth rate comparable to untreated cells. Whether this recovery is due to initiation of protective cellular mechanisms such as the release of GDNF, or to changes in the binding of toxic agents, needs further studying, particularly as a possible therapeutic concern [34].

This study reveals different responses to PD-CSF in each of two CNS cell types, with α-synuclein protein aggregations and redistribution leading to inevitable, eventual, cell death following disruptions in cell-cell communication due to loss of adhesion properties in microglia. In contrast, astrocytes, whose function is to provide endothelial support as part of the blood brain barrier, and transport fatty acids, do not succumb to PD-CSF exposure in such a deleterious and catastrophic manner and these diverse cellular responses show trends that, if properly delineated, might lead to the development of neuroprotective therapies, enhancing cell adaptation mechanisms and targeting the induction of protective and recovery pathways for improved cell survival outcomes. A role for α-synuclein in cell-cell communication and/or adhesion is implied by our findings, and provides support for the hypothesis of excess or abnormal accumulation of α-synuclein in the neurodegenerative process leading to disruption of microglia mediated cell signaling. Astrocytes on the other hand, appear to be more resistant to PD-CSF treatment, which may reflect a less critical role of α-synuclein in the normal functions of these cells, but indicates an important role in endothelial support.

## Methods

Microglia cells (human brain glioblastoma cells; ATCC Grade III tumor; #HTB15) were cultured and grown to confluency (3-5 days) in Dulbecco's Modified Eagles Medium (DMEM) containing 4 mM L-glutamine, 1.5 g/L sodium bicarbonate, 4.5 g/L glucose, 10% fetal bovine serum and 1% Penicillin/Streptomycin. Frozen cell stock was split 10 times and 6 plates from each split were used per experiment. Human astrocytes were obtained from fetal brain tissue (Clonexpress, Inc., Gaithersburg, MD) and grown to confluency (2-5 days) in 50:50 DMEM/F12 containing 5% fetal bovine serum, 1% Penicillin/Streptomycin, and 10 ng/ml of both epidermal growth factor and basic fibroblast growth factor. Astrocytes were split 5 times from frozen stock and 6 plates per split were cultured. Cells were washed with 1× phosphate buffered saline (PBS) and isolated with 0.25% trypsin/0.03% EDTA for subculturing. Cell lines were replenished with fresh media every 2 days or frozen in complete growth medium with 5% dimethyl sulfoxide for later use.

### Cerebrospinal fluid (CSF)

IRB approved informed written consent was obtained for CSF samples used in this study. CSF was obtained by lumbar puncture, collected in 2 ml tubes under sterile conditions, immediately placed on dry ice, then de-identified and stored at -80°. PD-CSF came from adult men and women (age range 55-73 years; n = 13; median age 64 years, 10 men and 3 women), who were in the off-medicine state (no PD medicines ≥ 12 hours) and who had carried the diagnosis of sporadic PD for over 7 years. The diagnosis was made by a Movement Disorders specialist [33]. Disease control CSF came from adult men and women (n = 5) with an age range of 45-68 year (median age 62 years; 4 men, 1 woman) who did not have PD and had no clinical or laboratory evidence of a neurodegenerative disease, or an active infectious, inflammatory process. Non-PD neurological disease controls included patients with spastic hypertonia from late stroke, hydrocephalus, or spinal cord injury (n = 5).

### Cell Growth, treatment, and cell count measures

Cells were split and grown to confluency in petri dishes containing glass cover slips, moved to dishes containing fresh culture medium and CSF was added to media at a ratio of 1:6, (1 ml CSF plus 6 ml media). On specified days, cover slips were removed from each treated group together with one untreated control, photographed using a Nikon Labphoto-2 microscope equipped with a MotiCam (Motic, Richmond, BC, Canada) at a magnification of 160× and placed on ice. Cell count stereology was via Microsoft Paint (Microsoft Corp., Redmond, WA, USA). Three fields from each treatment were imaged per experiment.

### Fluorescent staining

Cover slips were probed with Texas Red for α-Synuclein, DAPI for nuclei and FITC for actin (Invitrogen-Molecular Probes, Eugene, OR, USA). Immunofluorescence staining involved fixation in 3.7% formaldehyde, rinsing in 1× PBS, permeabilization in 0.5% Triton, followed by a 1× PBS rinse. The cover slips were incubated with 10% goat serum at 37°C for 45 minutes to decrease non-specific antibody binding. Samples were subsequently incubated with α-synuclein antibody in 10% goat serum for 30 minutes at 37°C. Secondary antibody, either a Rabbit polyclonal or a Mouse monoclonal ((Santa Cruz Biotechnology, Santa Cruz, CA, USA; Abcam, Cambridge, UK) was added to 10% Goat Serum in 0.05% Tween and incubated with the samples for 30 minutes at 37°C. Finally, the cover slips were counterstained with DAPI/FITC for 5 minutes.

### Deconvolution fluorescence microscopy

Deconvolution fluorescence microscopy and 3D image reconstructions of α-synuclein, actin and nuclei were generated as previously described [4,20,35]. Images were saved as TIFF files and treatment comparison statistical analyses (1 way ANOVA) of both cell count and α-synuclein densities (pixel numbers) were made using Corel ((Ottawa, Ontario, Canada) and SigmaStat software (SPSS, Chicago, IL, USA).

### Western blot analysis

Approximately 2.0 × 10^6 ^Human Astrocytes (Cat# HAST 040, Clonexpress, Gaithersburg, MD) and Human Glialblastoma cells, U-118 MG (Cat# HTB-15, ATCC, Manassas, VA) were treated with 300 μl of 1× sample buffer (2X = 10.0 ml of 10% SDS, 4.0 ml 1 M Tris-Cl pH = 6.8, 7.5 ml Glycerol and 45.5 ml ddH2O) diluted 1:1 in 1× PBS and boiled for 5 minutes. Samples were sonicated for 20 minutes then centrifuged for 10 minutes at 2000 rpm (Approximately 650-850 × g). Supernatant was collected and the pellet discarded. Protein concentrations were measured with a Pierce BCA Protein Assay Kit on a Molecular Devices Spectra Max 250 (Molecular Devices, Sunnyvale, CA) reading at 562 nm. Samples were separated on a 10% polyacrylamide gel (BioRad, Hercules, CA) in a BioRad Criterion apparatus, in 1× running buffer (10X = 30 g/L Tris, 144 g/L Glycine and 10.0 g/L SDS) at 60volts for 20 minutes, another 20 minutes at 80volts, and then 100volts for 1.5 hours on ice. Proteins from the gel were transferred to a PVDF membrane (BioRad) in 1× transfer buffer (10X = 30 g/L Tris and 144 g/L Glycine) at 60volts for 1 hour on ice. The membrane was blocked with casein (Rockland Blotto Dry Milk, Gilbertsville, PA) in 1× TBS buffer (10X = 2.0 g/L KCL, 30.0 g/L Tris Base) with 1.0 ml/L Tween-20 (TTBS) overnight at 4°C with shaking. The primary antibody, Mab for Alpha-Synuclein, clone-3H2897 (Cat# sc-69977, Santa Cruz Biotechnology, Santa Cruz, CA) was diluted 1:1000 in powdered milk/TTBS and incubated with the membrane on a rocker for 3 hours at 4°C. The membrane was washed 4 times in TTBS for 10 minutes with shaking then the secondary antibody, Goat anti-Mouse IgG-HPR (Cat# sc-2005 Santa Cruz Biotechnology) was diluted approximately 1:5000 in Casein/TTBS and incubated for 1.5 hours with shaking at 4°C. The membrane was again washed 4 times in TTBS for 10 min. with shaking, blotted dry and incubated in Pierce ECL Western Blotting Substrate (Product #32209, Thermo Scientific, Rockford, IL) at a 1:1 ratio of Luminol Enhancer:Peroxide Solution for 2 minutes, blotted dry and placed in a plastic bag. The membrane was exposed to Fisher B Plus Full Blue photographic film (Fisher, Hanover Park, IL) for 10 minutes, and then developed on a Kodak OptO Max 2000 Developer (Kodak, Rochester, NY).

## Authors' contributions

MCS made substantial contributions to the conception, design, acquisition of data, analysis of data, interpretation of data and drafting the manuscript as well as final revisions. JLB conceived the study, made substantial contributions to the study design, data acquisition and data interpretation. TME made substantial contributions to the data analysis, design and manuscript writing. BJP made substantial contributions to the acquisition of data, interpretation of data, analysis of data, study design and manuscript writing. KD made substantial contributions to the concept, analysis of data, acquisition of data and interpretation of data. RJB made substantial contributions to the study design, original concept, acquisition of data, interpretation of data, analysis of data and manuscript writing. All authors read and approved the final manuscript.
